# Functionality of Puff Pastry Olive Pomace Oil-Based Margarines and Their Baking Performance

**DOI:** 10.3390/foods12112138

**Published:** 2023-05-25

**Authors:** María Dolores Álvarez, Beatriz Herranz, Arancha Saiz, Susana Cofrades

**Affiliations:** 1 Institute of Food Science, Technology and Nutrition (ICTAN-CSIC), C/José Antonio Novais, 6, 28040 Madrid, Spain; a.saiz@ictan.csic.es (A.S.); scofrades@ictan.csic.es (S.C.); 2 Department of Food Technology, Veterinary Faculty, Complutense University, Avda/Puerta de Hierro, s/n, 28040 Madrid, Spain

**Keywords:** olive pomace oil, organogelator agents, puff pastry, bakery, rheological properties, healthy fat, thermal properties, sensory, laminated bakery product

## Abstract

Designing healthier lipids is a current approach to developing potential functional foods. Olive pomace oil (OPO) has beneficial effects on human health, attributed to its high oleic acid content and unique bioactive compounds. Four puff pastry margarines (PP-M), based on OPO (M1, M2 at 40.8%, and M3, M4 at 30.8%, and cocoa butter at 10%) combined with low molecular weight organogelators, were prepared using two initial cooling rates (M1, M3 at 0.144 °C/min and M2, M4 at 0.380 °C/min) and compared to both commercial puff pastry (PP) butter (CB) and fatty preparation (CFP). Subsequently, six baked PP counterparts were elaborated. Physical-chemical, mechanical properties, and lipid profiles were analyzed in M1–M4 and PP, while thermal properties were determined in M1–M4. Sensory analysis was carried out in PP-M1 and PP-M3 counterparts. Elasticity (*G*′) of M1–M4 samples was between that of controls CB and CFP, although a higher OPO content reduced viscous modulus (*G*″). The initial cooling rate did not affect the melting behavior of M1–M4. The firmness of PP-M1 was similar to that of PP-CB and PP-CFP, and the better spreadability and plasticity of M1 positively favored PP puffing. In addition, PP-M1 had 36.8% less SFA content than baked PP-CB, and its overall acceptability was similar. For the first time, a new margarine with high OPO content, showing adequate firmness, spreadability, and plasticity, was formulated, which gave rise to PP with appropriate performance and sensory quality and a healthy lipid profile.

## 1. Introduction

Spain is the world’s leading global producer of olive pomace oil (OPO). When olives are processed, only 20% of the oil extracted is olive oil (OO), while the remaining 80% is alpeorujo, or moist, fatty pomace, from which OPO is extracted. OPO has an excellent nutritional quality because it comes from the olive grove, but it also presents exceptional health and taste properties, and is both profitable and sustainable. In addition, although not widely consumed, OPO is a relevant source of dietary fat, with great health potential related to its composition, due to its high content in monounsaturated fatty acids (MUFA), mainly oleic acid, and unique minor bioactive compounds, such as squalene, pentacyclic triterpenes, tocopherols, sterols, and aliphatic fatty alcohols [1], which contribute to its health-promoting properties [2]. In a chronic clinical intervention study, OPO was shown to have hypolipidemic effects in both healthy subjects and in those with high blood cholesterol levels, contributing to the prevention of cardiovascular disease [3]. As well, two long-term intervention studies compared the health effects of OPO with those of high-oleic sunflower oil (HOSO, first study) and sunflower oil (SO, second study) in healthy individuals and in those at risk for cardiovascular disease [4]. These studies concluded that OPO is a healthier alternative to HOSO and SO for both groups of individuals. Therefore, the prolonged consumption of OPO has a beneficial effect on biomarkers that may have a positive impact on the vascular tone [2]. Moreover, OPO exhibits an excellent performance for frying, attributed to its smooth taste, resistance to thermal oxidation, stability of its minor bioactive compounds at frying temperature, and with twice the durability of SO [5,6]. In addition, in Spain, the pomace sector uses 100% of the by-products of the olive grove, transforming moist fatty pomace into OPO and other valuable products, such as biomass and compost [7]. The food industry demands technological processes that combine the aforementioned requirements, in terms of nutritional quality, profitability, and sustainability. In this sense, butter, shortening, and margarine face a challenge in the lipid industry, as they are the primary types of edible fats used to produce bakery products. Butter is highly valued for its characteristic flavor and aroma [8,9]; however, its major disadvantage is its high content of saturated animal milk fat (65% saturated fat) [8]. Recently, Álvarez et al. [9] reported that the lipid profile of a commercial PP fatty preparation (CFP) presented 51% less SFA and 60% more unsaturated fatty acids (UFA) than a commercial PP butter (CB). It is well known that excessive intake of saturated fat poses a serious threat to human health [10], and this overconsumption of saturated fat is leading to an increasing research interest in the use of structured healthy oils as an alternative to solid fat-based products. The functional properties of solid fat largely depend on the crystal network structure of the high melting point components in the fat [11]. An alternative to conventional saturated solid fat is the use of a structured three-dimensional network capable of trapping the liquid oil through the fat phase (FP) crystallization by organogelator agents, which are mainly the monoglycerides (MG) and diglycerides (DG) of fatty acids (FA) [9,12]. These agents are commonly obtained by interesterification of triglycerides (TG). In margarines and spreads, MG provides emulsification functionality [13]. 

Puff pastry (PP) is a very appreciated non-leavened bakery or pastry product with an airy, flaky appearance and distinctive textural properties conferred by its unique structure of alternating dough and fat layers [14,15]. Terms such as laminating fat, roll-in fat, or layering fat are commonly used to refer to the solid or semi-solid fat of PP [14]. In fact, the elaboration of PP is a very complex process, involving multiple production practices, and it remains an art [14]. For example, the uniqueness of the dough lift or lifting is related to the water present in the dough formulation. As the dough is baked, the water evaporates and tries to escape, while the fat layers trap the steam inside the dough, forcing the pastry to expand [15]. In turn, the height of the PP depends on the consistency of the multilayered structure of dough and fat layers, as well as their ability to maintain their structure during baking to achieve proper lift [16]. These factors are also highly dependent on the rheological properties of the laminating fat [15], which needs to be highly plastic and consistent to provide suitable functionality [16]. In this sense, the good performance of butter in PP is associated with the fact that the paste made with butter retains plasticity very well, leaving no gaps in the fat layer for air and steam to escape [17]. However, butter has an unsatisfactory spreadability at low temperatures [8]. On the other hand, while laminating fat plays a key role in the production of PP, reducing the fat and calorie content excessively is not feasible. Such a reduction would be in line with the increasing global trend towards healthier foods, but it would also have an adverse impact on the quality attributes of PP [18]. 

In turn, the rheological properties of layering fats depend on both the fat composition and the crystallization process [15], since they determine the fat crystal network properties, which include polymorphism. In addition, to obtain the best PP-M composition, it is necessary to study the compatibility, crystallization behavior, and phase behavior of the different oils and fats contained in the margarine formulation [19]. To control fat crystallization during the cooling process, commercial shortenings and margarines are mainly produced by rapid cooling with scraped surface heat exchangers (SSHE) [20]. It is well known that the *β*’ polymorph is the most desirable in margarines, due to its small crystal size, which confers a smooth mouthfeel and adequate spreadability [15,19,21]. In turn, a cooling temperature of 14–16 °C and a crystallization speed of 220–240 rmp were found to be optimal parameters for the formation of *β*’-crystals in margarine [21]. In general, fat polymorphism depends on both crystallization temperature and cooling rate, but cooling also influences crystal size [20,22].

A previous study [9] showed that, by using different combinations of organogelator agents, it is possible to formulate healthy PP-M, with OPO as the main ingredient. However, their physical characterization indicated that some improvements, mainly related to the increase in plasticity, were still necessary to achieve adequate shortening functionality and baking performance in PP. 

The scientific goal of this work was to study the relationship between the effects of composition and initial cooling rate on the physical functionality (physical-chemical properties and crystallization), as well as the FA profiles of PP-M containing OPO and PP counterparts together their baking performance. A sensory analysis was also carried out in PP samples. To this end, four margarines with a higher (M1 and M2) and a lower (M3 and M4) OPO content (41 and 31%, respectively) were prepared and statically crystallized at two different initial cooling temperatures: 20 °C in M1 and M3 and −24 °C in M2 and M4. Comparisons were made with a commercial butter (CB) and a commercial fatty preparation (CFP), which are commonly used as PP laminating fats.

## 2. Materials and Methods

### 2.1. Materials

The OPO used in this study was provided by Interprofesional del Aceite de Orujo de Oliva (ORIVA, Sevilla, Spain). Vandemoortele Europe NV (Ghent, Belgium) provided refined palm stearin (PS) flakes. Salted table butter from Hacendado (manufactured by COVAP, Córdoba, Spain) was purchased from a local supermarket. The emulsifiers, Verolec Non GMO IP, prepared with soybean lecithin (E-322), Verol N-90, distilled MG (E-471), Verol P, polyglycerol ester of FA (E-475), and beeswax were all provided by Lasenor (Barcelona, Spain). The emulsifier Palsgaard^®^ 1311, consisting of a blend of polyglycerol esters of FA and citric esters of MG and DG of vegetable FA, was provided by Palsgaard A/S (Juelsminde, Denmark). Barry Callebaut Manufacturing Iberica S.A.U. (Barcelona, Spain) supplied Barry white cacao pearls, which were employed as the cocoa butter source. Fine table salt (Aliada, Madrid, Spain), anhydrous citric acid (Panreac, Barcelona, Spain), ground dietary gelatin 200/220 Bloom from Manuel Riesgo S.A. (Madrid, Spain), butter flavor purchased from Arconsa S.A. (Murcia, Spain), and tartaric acid (Chefdelíce, Murcia, Spain) were also used. CB and CFP, both commonly used as laminating fats in PP, were acquired from Flechard SAS Laiterie Du Pont Morin (France) and St. Auvent^®^, Vandermoortele (Europe, Ghent, Belgium), respectively. In addition, for making PP doughs, strong flour for PP (Tradicional Zamorana, Zamora, Spain) and wheat flour (Gallo, Córdoba, Spain) were purchased from local retailers.

### 2.2. Margarine Preparation

Four margarines (M1, M2, M3, and M4) with two different fat phase compositions were prepared in batches of 1300 g. In all of them, the fat phase/aqueous phase (FP/AP) proportion was 80/20. The FP of M1–M4 contained the following fixed ingredients: PS 23.5%, table butter 7.5%, emulsifiers Verolec Non GMO IP 1.5%, Palsgaard 1311^®^ 1.17%, Verol N-90 0.83% and Verol P 1.75%, and beeswax 3%. The OPO content in margarines were 40.8% for M1 and M2, and 30.8% for M3 and M4, respectively. In addition, M3 and M4 contained 10% cocoa butter. The choice of the FP organogelators was based on the combinations and percentages recommended by the PP-M margarine manufacturers (Palsgaard^®^ and Lasenor), with minor modifications. The AP of all the margarines M1–M4 contained water 17.9%, salt 1.0%, citric acid 0.0025%, gelatin 0.50%, butter flavor 0.37%, and tartaric acid 0.25%, selected according the AP composition of the CFP, with slight modifications. The percentages given were calculated based on the total weight of the ingredients constituting both the FP and the AP. The composition of both the AP and FP in the formulated M1–M4 margarines is summarized in Table S1. 

To prepare the AP, all the ingredients were mixed and stirred using a magnetic stirrer at 300 rpm for 10 min at room temperature until completely dissolved. To prepare the FP, all the ingredients, with the exception of emulsifiers Verolec Non GMO IP, Palsgaard 1311^®^, and Verol N-90, were previously mixed together at 300 rpm with a rod stirrer (Bunsen AGV-8, Madrid, Spain) by placing them in a thermostatic water bath (Series BD, Bunsen, Madrid, Spain) at 70 °C. Then, the three emulsifiers were dissolved separately in one fifth of the FP at 70 °C, and were later mixed and homogenized with the rest of the FP. Once the FP was completely dissolved, it was introduced into a Thermomix^®^ glass at 60 °C. The emulsification process was performed at 50 °C in a Thermomix^®^ TM5-1 homogenizer (Vorwerk, Germany). The AP, which was at 12 °C, was incorporated into the FP at 1500 rpm for 3 min; then, the stirring speed was increased to 10,200 rpm, keeping this speed for 30 s. Next, the emulsion was cooled and stirred at 1500 rpm by placing it in a cold ice bath (5 °C) for 17 min. The final emulsion temperature was 39 °C. Each emulsion was then poured into plastic trays containing 330 g of sample, and two initial cooling rates for fat crystallization were tested. M1 and M3, without and with cocoa butter, respectively, were left to rest for 1 h at room temperature (cooling rate = 0.144 °C/min), and M2 and M4, with a similar composition to M1 and M3, respectively, were kept for 1 h at −24 °C (cooling rate = 0.380 °C/min). After the initial cooling, all the margarine samples were placed at a constant chilling temperature of 4 °C, to complete fat crystallization, and stored. As it was mentioned above, the four formulated margarines were referred to as M1, M2, M3, and M4, with both M1 and M3 having a slower initial cooling and M2 and M4 a faster one. The margarines or laminating fats were analyzed after 3 days of chilling storage at 4 °C.

### 2.3. Puff Pastry (PP) Elaboration

**Figure 1:**
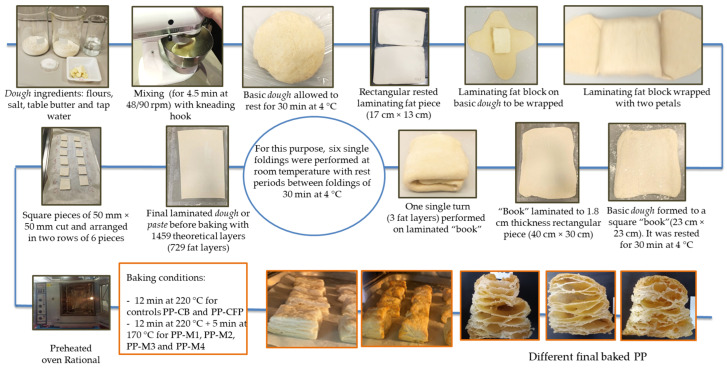
Puff pastry (PP) elaboration procedure.

Six baked PP were prepared using the four margarines (PP-M1, PP-M2, PP-M3, and PP-M4), mentioned above, and the two commercial PP products (PP-CB and PP-CFP). In accordance with Wickramarachchi et al. [14], in this study, the word “dough” refers to the basic mixture of flours, salt, table butter, and tap water, without the addition of margarine or laminating fat. The term “paste” refers to the mixture of dough and laminating fat before baking, while “pastry” or “PP” refer to the final baked product. To obtain PP, portions of laminated dough or paste were prepared according to the so-called French method [18,23], wherein a piece of each laminating fat (CB, CFP, M1–M4) was wrapped with basic dough and then folded several times to obtain a multi-layered dough or paste. The PP-making procedure used is shown in Figure 1. One day before making each PP, a rectangular fat piece or block (17 cm × 13 cm) of the corresponding laminating fat, representing 35.14% of the composition of the laminated dough, was sheeted to a thickness of 1.55 cm and left to rest for 24 h at 4 °C. The basic or initial dough, without the incorporated laminating fat, consisted of wheat flour (19.63%), strong pastry flour (19.63%), salt (0.4%), table butter (3.9%), and tap water (21.3%), which were mixed in a standard mixer (KitchenAid, Artisan, Mod. 5KSM150, Whirlpool Corporation, Greenville, Ohio, USA) with a kneading hook. Initially, both flours and the melted table butter were pre-mixed for 30 s at speed one (48 rpm). Then, water with diluted salt was mixed with the flours and the melted butter at speed 2 (90 rpm) for 4 min. This basic dough was covered with a plastic film to prevent drying and left to rest for 30 min at 4 °C. After that, the basic dough was kneaded by hand and then shaped into a “flower”, and the corresponding rested fat piece was placed on it and wrapped with the dough to form a 40 mm-thick square (23 cm × 23 cm), referred to as a “book”. The “book” was left to rest for 30 min at 4 °C, and then it was laminated to a 1.8 cm-thick rectangular block (40 cm × 30 cm) using a dough roller. Six single folds (729 fat layers) with the same dimensions were performed on this layered dough or “book” at room temperature, with rest periods of 30 min at 4 °C between foldings, which resulted in a laminated dough or paste with 1459 theoretical total fat and dough layers. After the last folding rest (30 min at 4 °C), the dough was sheeted to a rectangular block. This laminated dough was covered with plastic film and left to rest for 10 min at −20 °C. Then, square pieces of 50 mm × 50 mm were cut and arranged in two rows of 6 pieces on a tray with baking paper and baked in a preheated Rational oven (Combi-Master, CM6, Landsberg, Germany) to obtain each pastry or PP sample. The baking time was 12 min at 220 °C for controls PP-CB and PP-CFP, followed by an additional 5 min at 170 °C for PP-M1, PP-M2, PP-M3, and PP-M4.

### 2.4. Rheological Measurement of Margarine

Rheological characterization of the control laminating fats (CB and CFP) and formulated margarines M1–M4 was carried out with a Kinexus pro controlled-stress rheometer (Malvern Instruments Ltd., Worcestershire, UK), equipped with a serrated plate–plate geometry (Ø = 20 mm, 1.5-mm gap, ≈ 30 mL sample). The temperature was controlled at 20 °C with a high-temperature cartridge in the lower plate, and a cover cell to maintain the samples at the specific temperature and prevent evaporation. Before further testing, each sample was allowed to stabilize and restructure for 20 min using a time sweep test, performed at 1 Hz with a selected shear stress (*σ*) of 200 Pa within the linear viscoelastic region (LVR). Following, stress sweep tests were performed at 1 Hz with *σ* varying from 20 up to 2000 Pa, in order to determine LVR limit values. Then, frequency sweep tests (mechanical spectra) were carried out at 200 Pa within the LVR, ranging the frequency from 10 up to 0.1 Hz. From each test, elastic modulus (*G*′, kPa), viscous modulus (*G*″, kPa), and loss tangent (tan *δ* = *G*″/*G*′, dimensionless) values were recorded.

### 2.5. Oil Binding Capacity of Margarine

The oil binding capacity (OBC) was determined in accordance with Da Pieve et al. [24]. For that, 1 g of margarine or laminating fat was weighed and centrifuged at room temperature and at 10,000 rpm for 15 min, using a Thermoscientific centrifuge (Sorvall LINX 6000), to express the oil. The OBC was obtained from the percentage of oil released from each margarine after centrifugation.

### 2.6. Differential Scanning Calorimetry (DSC) of Margarine

The melting profiles of CB, CFP, and the formulated margarines M1–M4 containing OPO and PS, which was a major ingredient of control CFP and M1–M4 laminating fats, were measured using a differential scanning calorimeter (TA Q1000, TA Instruments, New Castle, DE, USA). Samples were prepared as previously described [9], equilibrated at −10 °C for 10 min, and heated from −10 to 80 °C at a constant rate of 5 °C/min. The melting peak temperatures were derived from the heating thermograms [9].

### 2.7. Color Measurement of Margarine 

Color measurements of the six laminating fats were carried out by reflectance (Chroma Meter CR 400, Konica Minolta Sensing, Inc., Osaka, Japan), using the CIE Lab scale (D65/10°). The parameters determined were L* (L* = 0 [black] and L* = 100 [white]), a* (−a* = greenness), b* (+b* = yellowness). The yellowness index (*YI*) was calculated as 142.86b*/L* [25] from the color parameters (L*a*b*), and the CIE76 formula [26] was used to calculate the color difference (ΔE*) between control CB and each of the other laminating fats. 

### 2.8. Texture Measurement of Margarine and Puff Pastry

A TA.HDPlus Texture Analyzer (Stable Micro Systems, Ltd., Godalming, Surrey, UK), equipped with Texture Exponent software (version 6.1.20.0) and a 5 kg load cell, was used. The texture of CB, CFP, and M1–M4 was instrumentally measured at 20 °C using a penetration test with a 4-mm cylindrical stainless-steel flat probe. The trigger force was 0.039 N, and the probe penetrated the sample to 10 mm at a rate of 1 mm/s. Force (N) at the final test (10 mm) and work (mJ), calculated as the area under the curve, were derived from the force-distance curves. For the baked PP piece counterparts or pastries, the texture was measured 1 h after baking using a texture profile analysis (TPA), based on a double cycle compression test. A flat 75-mm diameter aluminum plunger (P/75, SMS Ltd., Godalming, UK) was used. The test rate was set at 2 mm/s, and the waiting time between compression cycles was 3 s. The trigger force was 0.020 N. Each PP piece was compressed up to 30% of its initial height (30% strain), and, from the curve force-time generated by the TPA test, hardness (N), cohesiveness (dimensionless), and chewiness (N) were calculated as previously defined [27]. In addition, the number of force peaks, calculated for a drop in force higher than 0.05 N, was derived from the force-time profile corresponding to the first compression cycle of the TPA test. A cutting test was also performed on each PP by using a craft knife adapter (A/CKB; SMS Ltd., Godalming, UK), accommodating a standard 50 mm wide craft blade. The trigger force was 0.049 N. Each PP piece was cut through its center, up to a depth of 15 mm. The test rate was set at 2 mm/s, and cutting work (mJ), calculated as the area under the force-distance curve, was derived between the trigger force and force at the end of the test (15 mm). 

### 2.9. Fatty Acid (FA) Profile of Margarine and Puff Pastry

The FA contents of OPO, PS, CB, CFP, M1, and M3, as well as of the baked PP-CB, PP-CFP, PP-M1, and PP-M3 counterparts, were determined by saponification and methylation, as previously described [9,28]. Both the laminating fat and PP samples were lyophilized beforehand. Fatty acid methyl ester (FAME) was analyzed on an Agilent gas chromatograph (Model 7820A, CA, USA), fitted with a GC-28 Agilent DB-23 capillary column, and a flame ionization detector was used. Results are expressed as mg FA/g sample.

### 2.10. Baking Performance of Puff Pastry 

For all the six different PP produced, the weight (W_ld_) and height (H_ld_) of each square laminated dough piece with fixed surface area (50 × 50 mm^2^) were recorded beforehand. After baking and cooling to room temperature (~30 min), each PP piece counterpart was similarly weighed (W_PP_) and measured, and designated as PP or pastry lift. Weight loss (%) after baking was calculated as follows: (W_ld_ − W_PP_/W_ld_) × 100. In addition, other objective parameters commonly used in the evaluation of PP quality, such as specific lift, development, lift irregularity, and shrinkage, were calculated as described by Wickramarachchi et al. [14]. Measurements of the dimensions of both laminating doughs and PP were carried out manually with an electronic caliper. 

### 2.11. Sensory Analysis of Puff Pastry

The sensory panel consisted of 30 untrained panelists (19 female, 11 male), with ages ranging from 23 to 64 years old. The panelists were recruited from the ICTAN-CSIC staff, and, although they were not explicitly trained on PP sensory analysis, all had previous experience in performing sensory analysis of different foods. The evaluation was performed at room temperature in panel booths, in accordance with the International Standard [29]. The PP controls, prepared with CB and CFP, and the samples prepared with M1 and M3 margarines (having a different composition, i.e., without and with cocoa butter, respectively, and both initially cooled at 0.144 °C/min) were evaluated. The PP samples prepared with M2 and M4 were not tested, due to logistics reasons. After cooling (30 min after removing the samples from the oven), the samples were coded with a random three-digit number and presented simultaneously on a white plate in a randomized order. Each panelist evaluated the hedonic attributes “color”, “odor”, “taste”, “texture”, and “overall acceptability”, as well as the intensity of “fattiness” for each one of the 4 PP samples. A 10-point structured scale, ranging from 0 (dislike extremely/not intense) to 10 (like extremely/very intense) was used. Moreover, panelists were asked to indicate any unpleasant taste perception in comparison to the control samples PP-CB and PP-CFP.

### 2.12. Statistical Analysis

Analyses were conducted using the IBM SPSS software for Windows, Version 29.0 (IBM Corp., Armonk, NY, USA). All of the above measurements carried out on either laminating fat or PP samples were performed at least in triplicate. Evaluations of each formulated solid fat were conducted 3 days after formulation and storage at 4 °C, whereas each PP counterpart was prepared and evaluated one day after testing each laminating fat. Although all the different determinations carried out in controls CB and CFP are shown for comparison purposes, they were not included in the statistical analyses because the thermal history and process conditions of the control samples are likely to be very distinct and unknown to us. One-way analysis of variance (ANOVA) was used to compare the rheological, textural, color, and thermal parameters of the laminating fats or margarines, as well as the textural, performance quality parameters, and sensory attributes of the PP counterparts. Significant differences between pairs of means were evaluated by the Tukey test, using a 95% confidence interval (*p* < 0.05). 

## 3. Results and Discussion

### 3.1. Rheological and Textural Measurement of Margarine

**Figure 2:**
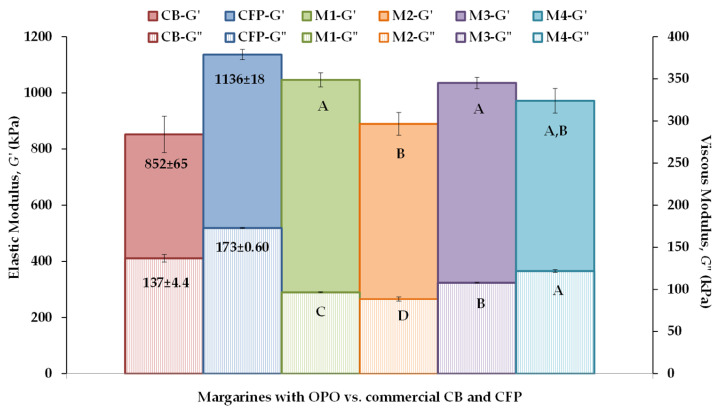
Viscoelastic moduli at 1 Hz and at 20 °C of a puff pastry (PP) commercial butter (CB), a PP commercial fatty preparation (CFP), and four margarines (M1–M4) containing olive pomace oil (OPO). *G*′, elastic modulus; *G*″, viscous modulus. ^A–D^ Effect of formulation and cooling. Different letters for the same viscoelastic property indicate significant differences (*p* < 0.05).

As an example, stress sweeps carried out to determine the LVR of CB, CFP, and the formulated margarines (M1–M4) are shown in Figure S1. The elastic modulus (*G*′) values of CFP overlap with those of M1, M3, and M4, although control CB and sample M2 had lower *G*′ values. In addition, both controls CB and CFP had higher *G*″ values than the formulated samples M1–M4, which would seem to indicate that these laminating fats have a stronger fat-crystal network than CB and CFP. CB and M2 exhibited a less compact and weaker network, with the lowest *G*′ values. However, the stress amplitude of 200 Pa was within the LVR of all the samples (Figure S1), reflecting a good similarity between the network structures of all six laminating fats. In addition, the *G*′ values, as well as the critical stress determined at the end of the LVR, were very similar to those previously reported in PP shortening made of palm oil and anhydrous milk fat blends [20,30]. Similarly, the mechanical spectra obtained for all six laminating fats are shown in Figure S2. In all samples, the *G*′ values were higher than those of *G*″ over the frequency (*f*) range analyzed, indicating that the crystal network acts as a viscoelastic solid. In addition, *G*′ exhibited higher *f* dependence than *G*″ in all laminating fats, which is consistent with previous findings in comparable PP solid fats [9]. Figure 2 shows the *G*′ and *G*″ values at 1 Hz derived from the mechanical spectra (Figure S2) for all six samples. In the six solid or hard fats, *G*′ was greater than *G*″, although the ratio between the elastic and viscous moduli was smaller in controls CB and CFP (≈6.5) than in the formulated samples M1–M4 (≈10). M2 had a *G*′ value significantly lower than that of M1 (Figure 2), indicating that margarine M2, which contained 40.8% OPO and was subjected to a faster initial static cooling (in a freezer), was softer than its M1 counterpart, which was statically cooled more slowly (at room temperature). In contrast, in PP-M prepared using palm oil and anhydrous milk fat blends, the samples that crystallized at a slower rate (20 °C) were softer than those that crystallized faster (12 °C). However, these differences diminished after one week of storage at 5 °C [20]. These authors attributed the results to the fact that more crystals are formed at colder temperatures, although they studied the effect of cooling on dynamic fat crystallization using an SSHE system, thus combining shear with rapid cooling [30].

In any case, in this study, it is possible to appreciate that the elasticity of samples M1–M4 (890–1046 kPa) falls within the range of values obtained for the two commercial controls. Nguyen et al. [20] previously reported comparable *G*′ values in the above-mentioned shortenings made of blends of palm oil, PS, and anhydrous milk fat (25% *w/w*), which were shear crystallized using a heat exchanger at 20 and 12 °C (980 and 1410 kPa, respectively). 

On the other hand, the *G*″ values of the four margarines containing OPO, ranging between 89 kPa for M2 and 122 kPa for M4, were lower than those of CB and CFP. This difference was more pronounced in M1 and M2, which contained 10% more OPO (40.8%). Therefore, a higher OPO content seems to reduce the viscous behavior. Consequently, the tan *δ* (= *G*″/*G*′) values of M1–M4, ranging between 0.0922 for M1 and 0.126 for M4, were also lower than in CB (0.152) and in CFP (0.162). This indicates that laminating fats containing more OPO have higher linear viscoelasticity (more solid behavior). The linear viscoelastic properties of fats are determined by the microstructure of the crystal network formed, which includes crystal aggregates differing in size (0.1 µm–140 µm) and morphology, held together by bonds of variable degree strength or reversibility [31]. It has also been found that, after nucleation, the crystal network of shortenings is a homogenous blend of tiny platelet-like crystals (<1 μm), mainly in the *β*’ form [20]. 

**Figure 3:**
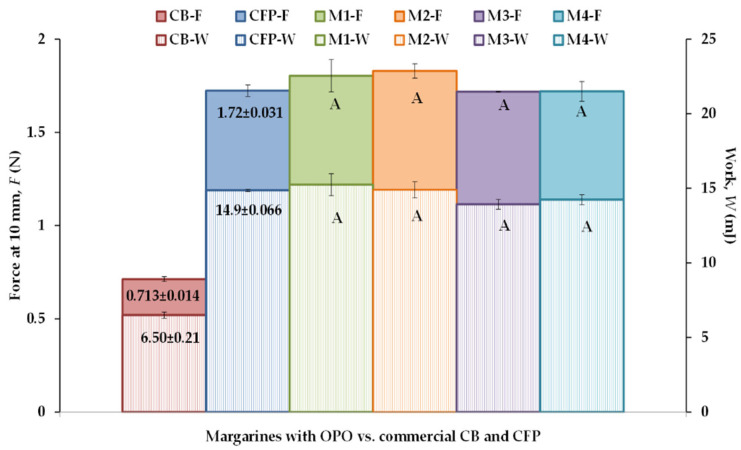
Textural parameters derived from penetration tests at 20 °C of a puff pastry (PP) commercial butter (CB), a PP commercial fatty preparation (CFP), and four margarines (M1–M4) containing olive pomace oil (OPO). ^A^ Effect of formulation and cooling. Different letters for the same mechanical parameter indicate significant differences (*p* < 0.05).

The end-uses of bakery fats involve significant, nonlinear deformations that are also related to the fat structure [31]. The nonlinear rheology of fats at large deformation can be measured from textural parameters derived by empirical methods, such as a penetration test. Figure S3 shows examples of force-distance curves obtained for all CB, CFP, and samples M1–M4 from a penetration test. A noticeable observation is that the force-deformation profiles for the control samples CB and CFP were slightly smoother, which would indicate better plasticity in the control fats than in the formulated M1–M4 fats [32]. However, there were no significant differences in the maximum penetration force and work values of samples M1–M4 (Figure 3). In addition, CFP had the same force value as M3 and M4, and the same work value as M2. Conversely, CB exhibited noticeable softening and higher spreadability (lower work value) at large deformations performed at 20 °C. This is associated with its high milk fat content (>82%), which is partly melted at this temperature [8] and must be kept cold to maintain its plastic behavior [17]. 

The correlations established between the rheological and textural data of all six laminating fats showed a negative significant (*p* < 0.05) correlation between both the penetration force (r = −0.680) and work (r = −0.579), and viscosity (*G*″ value). This was measured by small-strain amplitude oscillatory shear, and, therefore, without disturbance of the underlying fat crystal network. In contrast, correlations between both *G*′ and *G*″ were not significant. 

### 3.2. Oil Binding Capacity (OBC) and Color of Margarine

The OBC of the control fats CB and CFP, as well as that of all the formulated samples M1–M4, was zero. This indicates that there was no oil release due to the presence of a crystal network, mainly formed by MG and DG of FA. Moreover, in the case of M1–M4, this result also demonstrates the presence of an appropriate crystal size that could effectively trap and immobilize a high amount of OPO inside. 

**Figure 4:**
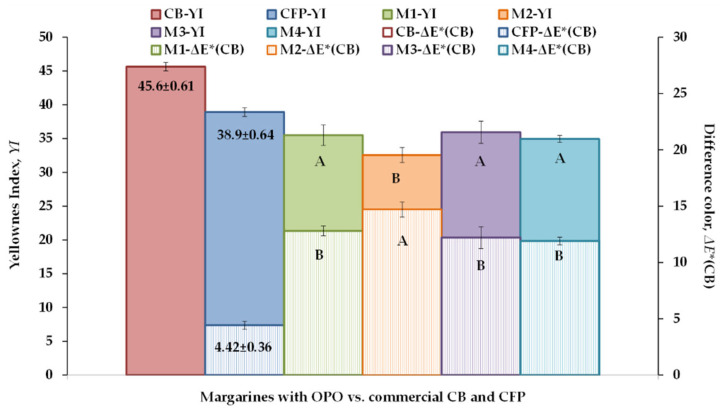
Color parameters of a puff pastry (PP) commercial butter (CB), a PP commercial fatty preparation (CFP), and four margarines (M1–M4) containing olive pomace oil (OPO). *YI*, yellowness index; ΔE*(CB), difference in color with CB. ^A,B^ Effect of formulation and cooling. Different letters for the same color parameter indicate significant differences (*p* < 0.05).

In turn, the calculated color parameters of laminating fats are presented in Figure 4. The yellowness index (YI) of M1–M4, which ranged between 32.6 and 35.5, was lower than that of both control samples CB and CFP, although there was also a notable difference between the YI value of both CB and CFP. On the other hand, the difference in color (ΔE*), relative to CB, varied between 11.9 and 14.7 in M1–M4 solid fats, and was also clearly higher than in control CFP. These color differences found among the laminating fats are evidently associated with the fat content and the FA composition of each one of them. As mentioned earlier, CB is a dairy product containing 82% milk fat, and the yellow color of this fat is attributed to vitamin A, carotenes, and other pigments [33]. The same authors also observed that an increase in butter fat content is directly related to an increase in YI. In contrast, although control CFP and samples M1–M4 had a high fat content (78 and 80%, respectively) and all of them included butter in their formulations, their fat phases were mostly of vegetable origin and were less yellowish. In any case, as the laminating fats were intended to be used in PP production, the observed color differences were expected to be masked after PP dough preparation and baking. 

### 3.3. Melting Behavior of Margarine

**Figure 5:**
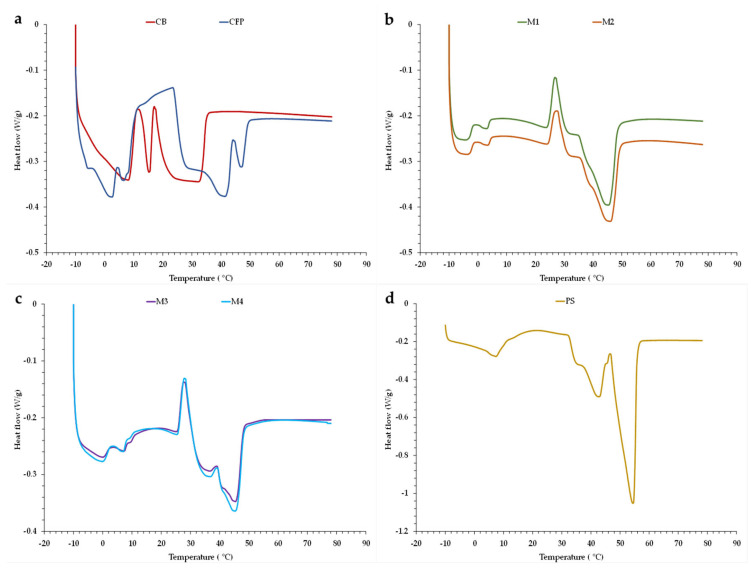
Melting profiles from DSC: (**a**) puff pastry (PP), commercial butter (CB), and PP commercial fatty preparation (CFP); (**b**) PP margarines (PP-M) containing 40.8% OPO (M1 and M2, initially cooled (1 h) at 20 and −24 °C, respectively); (**c**) PP-M containing 30.8% OPO and 10% cocoa butter (M3 and M4, initially cooled (1 h) at 20 and −24 °C, respectively); (**d**) palm stearin (PS).

The melting profiles of the control samples CB and CFP, as well as samples M1–M4 containing OPO and genuine PS, are shown in Figure 5. These profiles result from mixing solid fats and ingredients, liquid oils, and different emulsifiers; therefore, they exhibit similarities in certain aspects. In general, the melting profiles of all samples consist of two major parts, with a zone corresponding to high melting peaks, in the range of 40 to 50 °C (for CFP and M1–M4), and another zone related to low melting peaks, in the range of 0 to 10 °C. It is worth noting that the range of high melting peaks is lower for CB (Figure 5a) and higher for PS (Figure 5d). As previously reported [20,22,30], in control CFP (Figure 5a), the two higher melting peaks detected are associated with the presence of mainly fat crystals of palm oil and PS, including high melting TG, such as 1,3-dipalmitoyl-2-oleoylglycerol (POP), 2-oleoyl-1,3-rac-palmitoyl-stearoylglycerol (POS), and tripalmitoylglycerol (PPP). The melting of PPP crystals forms an endothermic peak in the range of 40–55 °C [20], and it is a major TG in PS, causing the distinctive second and third melting peaks of its melting profile (Figure 5d). However, POP and 1,2-dioleoyl-3-palmitoylglycerol (POO) are completely melted above 40 °C [30]. In addition, it is known that CFP contains PS among their ingredients (quantity is not indicated by the manufacturer). For this reason, and probably due to a dilution effect, the third distinct peak of PPP (50–55 °C) in PS (Figure 5d) is converted in a sub-peak in CFP (Figure 5a), disappearing in M1 and M2 (Figure 5b), as well as in M3 and M4 (Figure 5c), as compared to CFP. When the melting point between components of fat blends is above 20 °C, TG mixtures often show a monotectic behavior or a dilution effect [22].

In the case of CFP, the low melting peaks mainly correspond to the low melting TG from palm oil, such as POO and 1,2-dipalmitoyl-3-oleoylglycerol (PPO), and from milk fat TG, which were present either in CFP or in M1–M4, and with a carbon atom number of C36–C42 [22], i.e., mostly medium molecular mass TG [34]. On the other hand, control CB exhibited a melting curve characteristic of milk fat, with three endothermic peaks between 0 and 10 °C, around 15, and between 20 and 40 °C (Figure 5a), which correspond to the low, medium, and high melting point fractions of TG, respectively. Fatouh [35] also observed three endothermic peaks in the DSC melting profile of milk fat, which were attributed to the melting of TG with different melting points. In addition, the proportion of saturated TG (C30:0~C50:0) is higher than that of unsaturated TG in milk fat [34].

On the other hand, fat polymorphism has been reported to depend on both crystallization temperature and cooling rate [20,30]. However, in this study, the two different initial cooling rates (0.144 and 0.380 °C/min) or crystallization temperatures (at 20 and at −24 °C) used did not significantly affect the melting behavior of the margarines M1 vs. M2 (Figure 5b) and M3 vs. M4 (Figure 5c). Perhaps these conditions were applied for an excessively short time (1 h) to generate differences in fat polymorphism. The effect of much higher cooling rates (29 °C/min and 44 °C/min) on fat crystallization of PP butter was also studied, and no polymorphic differences were observed between samples [36]. In accordance with Nguyen et al. [20], polymorphic transitions between crystals occur during extended isothermal stages.

The melting peak temperatures (T_mp_) derived from the thermograms are shown in Table 1. As indicated above, three T_mp_ values corresponding to each TG fraction were detected in control CB at 8.40, 15.2, and 33.3 °C, respectively, which confirms previous findings during the melting of milk fat [34]. These endothermic peaks are strongly associated with small, medium, and large molecular mass TG, respectively. Furthermore, 3 endothermic peaks were detected in PS, and these peaks shifted towards lower temperatures in CFP and M1–M4 laminating fats, all of them containing PS. In addition, in margarines M1–M4 containing OPO, 3 endothermic peaks were also detected, whereas 4 peaks were identified in CFP (Table 1). In both CFP and M1–M4, this first lower peak (T_mp1_) could be related to the different monoglycerols (MG) and respective amounts used as emulsifiers in control CFP and in M1–M4 [37]. The second peaks (T_mp2_) of CFP are associated with olein and unsaturated TG, such as POO and palmitoyl-oleoyl-linoleoyl-glycerol (LOP) [22,37]. As for the TG composition of crude OPO, the contents of POP, POS, and SOS, as target TG, were reported to be 11.0, 20.0, and 11.7%, respectively [38]. However, their T_mp2_ values could be related to the presence of other low melting point TG in OPO. Finally, T_mp3_ and T_mp4_ in CFP, as well as T_mp3_ in M1–M4, are attributed to the stearin fraction and its saturated TG, such as POP, PPP, and 1,3-palmitoyl-2-linolenoylglycerol (PLP). The findings of this study are in agreement with those reported by other researchers [9,37].

### 3.4. Fatty Acid (FA) Profile of Margarine and Puff Pastry

The FA profiles of OPO and PS, as the major ingredients of formulated fat blends M1–M4, as well as those of controls CB and CFP, and of the formulated samples M1 and M3 (without and with added cocoa butter, respectively), are shown in Table S2. The lipid compositions of M2 and M4 were expected to be similar to those of M1 and M3, respectively, because they possess identical compositions. A statistical comparison of the fat composition was not performed, due to significant differences in both saturated and unsaturated FA (UFA) contents. 

OPO has an excellent low (0.184) SFA-to-UFA ratio (Table S2), which is very similar to that reported in soybean (0.164) and sunflower (0.153) oils [22]. In contrast, and according to the same authors, the ratio is 1.21 in palm oil, which is present in the formulation of control CFP. With a content of 112 mg FA/g Sample (11.8% of its fat composition), and representing the 76.2% of its SFA, palmitic acid (C16:0) is the major saturated FA in OPO, followed by stearic acid (C18:0) (18.2% of total SFA). As expected, oleic acid (C18:1n9) is the most abundant MUFA in OPO (69.0% of its fat composition), followed by PUFA, such as linoleic acid (C18:2n6) (11.4% of its fat composition). A very similar fat composition was previously reported for OPO from three different batches supplied by ACESUR (Acesur, SA, Sevilla, Spain) [5], with C18:1n9 as the major FA (72.0–73.8%), followed by C16:0 (11.0–11.5%), and C18:2n6 (9.54–11.1%). Holgado et al. [5] also reported OPO trans FA levels below 0.32%. As shown previously [9], PS has a particularly high SFA-to-UFA ratio (3.14). This is undesirable from a health standpoint, but exhibits a crystallization behavior similar to that of palm oil, imparting plasticity and body to margarine fats [37], due to its high C16:0 content, which represents 87% of its SFA (Table S2). Similar results were reported by Saadi et al. [39] for PS, with a 57.5% of C16:0 and a 64.9% of SFA on total FA (%). 

In this study, M1 and M3 were formulated using OPO as a major ingredient (at 40.8 and 30.8%, respectively), in combination with PS (at fixed 23.5%) and other commonly used organogelator agents rich in MG and DG. While CFP had 49% less SFA content than CB (278 vs. 563), M1 and M3 had 37% and 28% less saturated fat than control CB, respectively (Table S2). The higher C16:0 content of M1 and M3 compared to CFP is attributed, at least partially, to the beeswax present in their formulation. The higher SFA content of M3 compared to M1 is due to its fixed 10% cocoa butter content. A previous study also showed that cocoa butter is high in SFA (63% of total FA), with C18:0 and C16:0 being its major saturated FA [40]. In CB, CFP, M1, and M3, the saturated fat is located in different TG structures. In control CFP, M1 (PS:OPO 23.5:40.8), and M3 (PS:OPO:cocoa butter 23.5:30.8:10), the principal saturated TG are derived mainly from PS containing PPP [17], as well as PPO/POP [37,41]. 

As a result, M1 and M3 had a much lower SFA content (41.1% and 46.9%, respectively) than control CB (74.1%). According to Garcia-Macias et al. [17], a PP with good physicochemical and sensory properties can be prepared using fat blends containing 50% SFA content. M1 (and M2), as well as M3 (and M4), had a lower SFA content, which is in line with the recommended healthy intake of saturated fat. In this recommendation, it is advised that SFA intake should not exceed 10% of total energy intake [10]. 

In turn, the FA composition of baked PP made with control CB and CFP, as well as fat blends M1 and M3, is shown in Table 2. In the presence of the remaining dough ingredients (Figure 1), flours at 39.3% and table butter at 3.9%, PP-CFP and PP-M1 had the highest and lowest C16:0 contents, respectively, and PP-M3 had the highest C18:0 content. However, both PP-M1 and PP-M3 had lower SFA contents. Compared to control PP-CB (Table 2), baked PP containing M1, M3, and CFP presented a reduction in SFA content by 36.8%, 24.8%, and 11.2%, respectively. In addition, C18:1n9 content was also higher in PP-M1 and PP-M3 compared to both controls PP-CB and PP-CFP. As a result, the SFA-to-UFA ratio in final baked pastries was 2.62, 1.29, 0.799, and 1.00 in PP-CB, PP-CFP, PP-M1 (or PP-M2), and PP-M3 (or PP-M4), respectively. Clearly, it is possible to benefit from the fact that PP-M1 and PP-M3 are much healthier than controls PP-CB and PP-CFP because they contain a noticeably lower amount of saturated fat (Table 2). Furthermore, using a by-product of the olive grove adds value to the product.

### 3.5. Texture of Puff Pastry 

Figures S4 and S5 show examples of force-time and force-distance profiles derived from the TPA and cutting tests. In all the samples, during the first compression cycle of the TPA test (Figure S4), as the plunger compressed the PP piece, the flaky and layered structure of the product fractured, resulting in a jagged profile. However, all the profiles for the second compression of the margarine samples were smooth, thus indicating that the fracture of these delicate structures occurred during the first deformation (up to 30%). In contrast, in the cutting test, all the complete profiles were jagged (Figure S5). This jaggedness results from shearing with nearly no compression of the layered structure. Table 3 shows the textural properties and parameters derived from both the TPA and cutting tests for all baked PP samples. Among the PP prepared with M1–M4, PP-M1 and PP-M2 had the lowest and the highest hardness, respectively, whereas PP-M3 and PP-M4 exhibited intermediate hardness. However, the hardness of all the PP with M1–M4 was higher than that of both controls PP-CB and PP-CFP. A priori, this result could indicate that the baked dough layers in PP with margarines containing OPO were slightly thicker, and, consequently, more force was needed to compress the pieces.

In contrast, all the PP samples made with M1–M4 had similar cohesiveness, which was also comparable to that of PP-CB and PP-CFP. This implies that all the PP withstood the second deformation, relative to their resistance under the first deformation, in a similar way [27]. Chewiness from the TPA, which represents the force, work, and distance required to compress each PP piece, was also significantly (*p* < 0.05) lower in PP-M1 than in the PP prepared with laminating fats M2-M4, and also higher than the chewiness of PP-CB and PP-CFP. This result could be expected, as hardness is involved in the chewiness calculation [27]. Additionally, from the TPA test, the PP crispy character was measured as the number of force peaks registered during the first compression cycle (Figure S4). This textural parameter was quite similar in all the PP-samples (Table 3), although, in the PP prepared with M1–M4, it was even higher than in those prepared with CB and CFP.

In turn, cutting work (total firmness) is another important parameter that describes the texture and internal structure of PP [23]. As for hardness, PP-M1 showed a significantly (*p* < 0.05) lower cutting work value than the PP made with the other fats M2-M4. This indicates that more force was needed to cut and penetrate PP-M2, PP-M3, and PP-M4. Interestingly, the total firmness value of PP-M1 was intermediate between those of controls PP-CB and PP-CFP. In PP-M3 and PP-M4, the higher hardness and cutting work might be attributed to the reduction of OPO (30.8%) in M3 and M4, as compared with M1, with 40.8% OPO, and to the fact that both M3 and M4 contained cocoa butter (10%) in their formulation. In this study, a significant positive correlation (r = 0.815) was established between the hardness and cutting work values of the laminating fats. Cocoa butter itself presents a strong and cohesive internal network, with high connectivity [28] and viscoelastic properties at 1 Hz and 20 °C that are significantly higher than those obtained under identical conditions for the laminating fats (Figure 2). Additionally, in this study, all the laminating fats were stored in refrigeration (4 °C), and the basic doughs and pastes were allowed to rest for periods of 30 min at 4 °C between foldings (Figure 1). Compared with 20 °C, an increase in connectivity, viscoelasticity, and consistency of M3 and M4 could be expected at 4 °C, in response to the more complete fat crystallization of cocoa butter fat crystals observed at lower temperatures [28]. A crystallization completion temperature of 6.3 °C, and an onset melting one of 30.6 °C, have also been reported for cocoa butter [42]. Therefore, during the lamination procedure, cooling at 4 °C could have a negative effect on the spreadability of margarines M3 and M4 and, consequently, have a negative layering effect on either the texture or the performance of the PP samples made with them. After refrigeration at 4 °C, the hardness or consistency of these margarines was excessive for proper handling and lubrication of the dough layers during sheeting. PP laminating fat must keep the dough layers separated throughout the folding process [17]. In this context, when insufficiently plastic M3 and M4 were used, gaps may have remained in the fat layers, facilitating air leakage, and detrimentally affecting performance.

In the case of the PP elaborated with M1 and M2, both with a higher OPO content (40.8%), PP-M2 showed significantly higher hardness, chewiness, and cutting work than PP-M1. However, PP-M1 had a total firmness (cutting work) value very similar to those of both controls (PP-CB and PP-CFP). Therefore, a faster initial cooling rate could promote a harder pastry, forming a more disordered and unstable network of fat crystals at 24 °C, due to the rapid formation of bonds. This would result in a harder-to-chew PP, as happened in PP-M2. It is also likely that fewer fat layers were formed in this baked pastry, as the laminating fat M2 showed lower elastic and viscous moduli than M1, M3, and M4 (Figure 2). However, in the case of the PP made with a lower OPO content (M3 and M4), there were no differences in these mechanical parameters, even though their initial cooling rate was also different. This discrepancy could be due to the fact that M3 and M4 contained 10% cocoa butter in their formulations, as explained earlier.

### 3.6. Performance of Puff Pastry

The performance of the PP prepared with controls CB and CFP and M1–M4 laminating fats is presented in Table 4.

The baked products made from M3 and M4 showed a significantly (*p* < 0.05) higher loss of weight relative to the laminating dough weight, compared with the PP made from M1 and M2. However, the weight loss in all four cases was higher than that of the control PP prepared with CB. As observed in this study, the good performance of butter in PP, with the lowest weight loss, has been associated with the fact that the pastry prepared with butter maintained its plasticity, thus preventing disruptions in the fat layers that promote air escape during baking [17]. When considering PP or pastry lift as the measurement of PP height [14], PP-M1 and PP-M3 had more height than PP-M2 and PP-M4, evidencing that slower initial fat crystallization produced better spreadability during the folding process. As a result, more impervious fat layers contributed to both air and steam trapping during baking. The build-up of steam pressure causes the dough layers to separate, which determines PP lift [14]. In this study, the use of control CB in laminated dough or paste also resulted in better puffing of the PP or pastry during baking. A solid fat content of 38%–45% at 20 °C resulted in the maximum-specific PP height [43]. The specific height of the PP was also strongly influenced by water absorption and extensibility of the dough, which is desirable to enable paste to withstand sheeting and avoid rupturing of the laminates and tearing of the dough sheets [44].

The specific lift of PP-M2 was significantly lower compared with the other PP made with margarines containing OPO, while the control PP CFP showed the highest specific lift (Table 4). This quality criterion of PP performance is calculated as the PP-lift-to-paste-weight ratio, and it is considered a better objective parameter to compare PP made from different paste thicknesses/weights than PP lift [14]. In this study, the paste weight was higher in M1 and M2 (17.5 ± 1.7 and 18.1 ± 1.5, respectively) than in M3 and M4 (13.9 ± 1.4 and 14.4 ± 1.8, respectively), with values of 16.4 ± 1.1 and 14.4 ± 2.0 in CB and CFP pastes, respectively. For example, these differences in paste weight caused PP-CB, with higher lift, to have lower specific lift than PP-CFP (Table 4). The PP-making procedure with all laminating fats was identical, with the same weight of ingredients and number of dough and fat layers. Therefore, the differences observed in paste weight and thicknesses of CB, CFP, and M1–M4, as well as in PP lift, can be partially attributed to the inherent experimental variability in the manually cut, laminated dough pieces (50 mm × 50 mm). Moreover, the better spreadability and plasticity of controls CB and CFP, as well as of M1, led to a more homogeneous fat layer distribution between the dough layers, which also favored PP puffing. The development, in turn, is the ratio between the PP lift and the height of the paste before baking [45]. In this study, this objective parameter was significantly higher in PP-M1 than in pastries made with M2-M4 (Table 4). Bakers typically expect the PP development to be between 5 and 8 [14]. According to professionals, the development after baking should be almost five times the initial paste height [45], but margarines M2 and M4, both with faster initial cooling and with worse plasticity, did not meet this requirement. Lift irregularity measures PP aesthetic quality as the difference between the maximum and minimum PP heights [14], and the higher irregularity values corresponded to the PP made with M3 and M2, whereas the pastries with M1 and M4 had similar lift irregularity to control PP-CB. Finally, shrinkage measures PP contraction after baking, and the highest shrinkage values were obtained in PP-M2 and control PP-CFP, reflecting higher PP deformation. Undesirable shrinkage and tearing of the dough during lamination can be caused by inadequate relaxation of the dough and/or paste during the resting periods. For example, the toughness and resistance to stretching of wheat dough decreases over time after mixing, reflecting a continuing change in the gluten structure, with a decrease in elasticity. The extent of dough shrinkage has also been directly related to the fat content of the samples [46].

The hardness or force required to compress PP-M2, PP-M3, and PP-M4, as well as the cutting work to shear them, were high (Table 2), indicating that these baked products were too hard. This is likely related to a worse performance, with lower PP lift and development values and higher lift irregularity values. All the doughs and pastes were allowed to rest at 4 °C during 30 min between the folding and sheeting steps to prevent temperature elevations in the paste and reduction in baking quality. However, it can be concluded that M3 and M4 would not need cooling at 4 °C during PP preparation, since it negatively affects their spreadability. In addition, it would be preferable to store these laminating fats at 12–18 °C to prevent excessive firmness from cocoa butter hardening, due to recrystallization of cacao fat crystals.

### 3.7. Sensory Analysis of Puff Pastry

**Figure 6:**
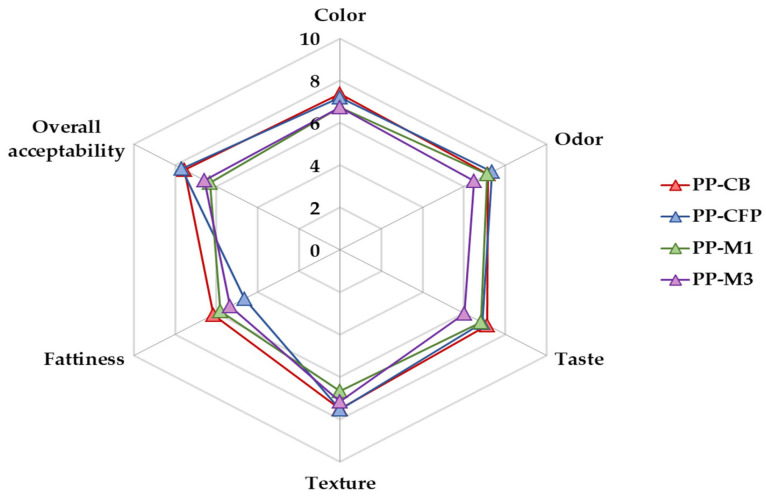
Sensory properties of puff pastry (PP) made with a commercial butter (PP-CB), a commercial fatty preparation (PP-CFP), and two margarines (PP-M1/PP-M3) containing olive pomace oil (OPO).

Figure 6 shows the mean scores given by 30 untrained panelists to the perceived sensations, attributes, and overall acceptability of the four baked PP samples. The mean scores for color, odor, taste, texture, and overall acceptability were high, ranging between 6 and 8 on a scale of 10. However, the scores for fattiness were lower, and ranged between 4 and 6. Notably, the scores for color, odor, taste, and overall acceptability were slightly lower in PP-M1 and PP-M3, compared to PP-CB and PP-CFP, and the score for texture was also slightly lower in PP-M3. Meanwhile, the scores for fattiness in PP-M1 and PP-M3 were between those given by panelists to PP samples made with control CB and CFP. However, there were no significant differences in the sensory attributes or overall acceptability of the four PP samples. As for the overall acceptability of the PP, it decreased in the order PP-CFP > PP-CB > PP-M3 > PP-M1, with the highest and lowest overall scores being 7.67 and 6.59, respectively (Figure 6). Holgado et al. [5] reported similar results for the sensory properties of fried potatoes using different OPO for frying, as compared to SO and HOSO. None of the attributes tested (color, oiliness, texture, and taste, as well as global appreciation) showed significant differences between the potatoes fried in the different oils. In this study, the results suggest that it is possible to replace both controls CB and CFP solid fats with either M1 or M3, containing 40.8 and 30.8% of OPO, respectively, to produce baked PP without affecting overall acceptability. Another sensory acceptance test also showed that laminating fat could be reduced by 36%, without adversely affecting the PP product, when compared to conventional products with a high fat content (33 wt %) [18].

## 4. Conclusions

The rheological measurements indicate that all six laminating fats had similar fat crystal network structures, although those containing OPO (M1–M4) were more viscoelastic than controls CB and CFP. A higher initial cooling rate (M2 and M4) tended to decrease the elastic modulus (*G*′) and increase the loss factor (*G*″/*G*′), compared with a slower initial cooling (M1 and M3). In addition, M1–M4 had the same textural parameters as CFP, whereas CB exhibited softening and higher spreadability at 20 °C. The firmness of baked PP made with M1 was similar to that of PP containing controls CB and CFP, while the highest hardness of PP-M3 and PP-M4 was attributed to a lower OPO content (30.8%) and a complete crystallization of cocoa butter fat crystals at 4 °C. Cooling at 4 °C during lamination negatively affected the spreadability of M3 and M4, resulting in a negative layering effect that influenced either the texture or performance of the baked PP. OPO has an excellent low SFA-to-UFA ratio (0.184), and, consequently, M1/M2 and M3/M4 had 37% and 28% less saturated fat than control CB, respectively. Results also evidenced that it is possible to replace both the control CB and CFP laminating fats with either M1 or M3, containing 40.8 and 30.8% OPO, respectively, to produce baked PP, without affecting the overall acceptability. Although substituting saturated fat is very challenging, as it provides unique characteristics, the formulated margarine M1 with high OPO content (40.8%) showed adequate firmness, spreadability, and plasticity. Therefore, the PP elaborated with this margarine had a similar performance and sensory quality to those elaborated with animal or milk fat.

## Figures and Tables

**Table 1:** Melting peak temperatures of a puff pastry (PP) commercial butter (CB), a PP commercial fatty preparation (CFP), and four margarines (M1–M4) containing OPO and palm stearin (PS).

	*T*_mp1_ (°C)	*T*_mp2_ (°C)	*T*_mp3_ (°C)	*T*_mp4_ (°C)
CB	8.40 ± 0.098	15.2 ± 0.023	33.3 ± 0.24	-
CFP	2.56 ± 0.050	8.33 ± 0.030	41.8 ± 0.12	47.3 ± 0.070
M1	−3.20 ± 0.11 ^C^	3.44 ± 0.30 ^B^	46.3 ± 0.87 ^A^	-
M2	−2.75 ± 0.12 ^B^	3.54 ± 0.15 ^B^	46.6 ± 0.37 ^A^	-
M3	0.150 ± 0.00 ^A^	7.31 ± 0.076 ^A^	45.8 ± 0.16 ^A^	-
M4	0.290 ± 0.026 ^A^	7.25 ± 0.20 ^A^	45.9 ± 0.24 ^A^	-
PS	7.54 ± 0.036	43.0 ± 0.13	54.6 ± 0.10	-

Mean values (*n* = 3) ± standard deviation. CB, commercial butter; CFB, commercial fatty preparation; M1–M4, margarines containing olive pomace oil (OPO); PS, palm stearin; *T*_mp1_, *T*_mp2_, *T*_mp3_, *T*_mp4_, melting peak temperatures. ^A–C^ Effect of formulation and cooling. Different letters in the same column for the same thermal parameter indicate significant differences (*p* < 0.05).

**Table 2:** Fatty acid (FA) profiles corresponding to baked pastries elaborated with a puff pastry (PP) commercial butter (PP-CB), a PP commercial fatty preparation (PP-CFP), and two margarines containing OPO (PP-M1 and PP-M3).

Fatty Acids (mg FA/g Sample)	PP-CB	PP-CFP	PP-M1	PP-M3
Butyric C4:0	4.67 ± 0.17	0.461 ± 0.014	0.710 ± 0.032	0.708 ± 0.0090
Caproic C6:0	5.03 ± 0.19	0.534 ± 0.012	0.803 ± 0.031	0.820 ± 0.0076
Caprylic C8:0	4.06 ± 0.13	0.603 ± 0.081	0.757 ± 0.015	0.782 ± 0.0017
Capric C10:0	10.3 ± 0.32	1.38 ± 0.019	1.96 ± 0.023	2.06 ± 0.0072
Lauric C12:0	13.7 ± 0.43	2.66 ± 0.028	2.66 ± 0.026	2.81 ± 0.013
Myristic C14:0	43.0 ± 1.3	8.60 ± 0.096	8.49 ± 0.067	8.91 ± 0.042
Pentadecylic C15:0	4.55 ± 0.14	0.703 ± 0.0074	0.839 ± 0.0071	0.871 ± 0.033
Palmitic C16:0	131 ± 3.9	188 ± 1.09	120 ± 1.0	132 ± 0.58
Margaric C17:0	2.45 ± 0.070	0.626 ± 0.072	0.635 ± 0.085	0.719 ± 0.0045
Stearic C18:0	38.2 ± 1.1	23.9 ± 0.30	23.5 ± 0.17	67.7 ± 0.123
Arachidic C20:0	0.595 ± 0.0070	1.47 ± 0.016	1.48 ± 0.014	1.88 ± 0.071
Behenic C22:0	0.221 ± 0.013	0.3017 ± 0.0048	0.573 ± 0.0086	0.587 ± 0.0027
Lignoceric C24:0	0.158 ± 0.046	0.310 ± 0.077	0.846 ± 0.096	0.751 ± 0.0063
**∑SFA**	**258**	**229**	**163**	**194**
Myristoleic C14:1n5	3.89 ± 0.12	0.439 ± 0.0054	0.660 ± 0.0048	0.717 ± 0.0048
Palmitoleic C16:1n7	6.18 ± 0.20	1.16 ± 0.014	2.64 ± 0.025	2.52 ± 0.012
Vaccenic C18:1n7	1.88 ± 0.049	2.46 ± 0.023	4.66 ± 0.052	4.10 ± 0.023
Oleic C18:1n9	75.6 ± 2.26	135 ± 1.4	163 ± 1.5	156 ± 0.80
**∑MUFA**	**87.6**	**139**	**171**	**163**
Linoleic C18:2n6	9.19 ± 0.27	37.45 ± 0.35	31.8 ± 0.29	29.2 ± 0.14
Linolenic C18:3n3	1.82 ± 0.047	1.18 ± 0.011	2.00 ± 0.022	1.87 ± 0.0088
**∑PUFA**	**11.0**	**38.5**	**33.8**	**31.1**
**∑MUFA + ∑PUFA**	**98.6**	**178**	**204**	**194**

Mean values (*n* = 3) ± standard deviation. SFA, saturated fatty acids; MUFA, monounsaturated fatty acids; PUFA, polyunsaturated fatty acids.

**Table 3:** Textural parameters derived from the texture profile analysis (TPA), and cutting tests of puff pastries (PP) prepared with margarines containing OPO, in comparison with PP elaborated with controls CB and CFP.

PP	Hardness (N)	Cohesiveness (−)	Chewiness (N)	Force Peaks Number (−)	Cutting Work (mJ)
PP-CB	3.62 ± 0.28	0.248 ± 0.023	0.525 ± 0.086	4.67 ± 1.2	28.2 ± 1.6
PP-CFP	3.79 ± 0.35	0.228 ± 0.018	0.387 ± 0.051	5.00 ± 1.0	31.7 ± 0.69
PP-M1	6.58 ± 1.2 ^C^	0.220 ± 0.035 ^A^	0.625 ± 0.18 ^B^	7.00 ± 1.0 ^A^	30.4 ± 3.5 ^B^
PP-M2	13.6 ± 0.91 ^A^	0.243 ± 0.016 ^A^	1.56 ± 0.24 ^A^	5.67 ± 0.58 ^A^	51.5 ± 1.5 ^A^
PP-M3	8.72 ± 0.77 ^B,C^	0.235 ± 0.018 ^A^	1.16 ± 0.11 ^A^	5.33 ± 0.58 ^A^	44.1 ± 5.4 ^A,B^
PP-M4	10.6 ± 0.89 ^B^	0.223 ± 0.0035 ^A^	1.18 ± 0.13 ^A^	6.33 ± 0.58 ^A^	50.9 ± 8.1 ^A^

Mean values (*n* = 3) ± standard deviation. PP-CB, PP made with a commercial butter; PP-CFB, PP made with a commercial fatty preparation; PP-M1, PP-M2, PP-M3, and PP-M4, PP prepared with four margarines containing olive pomace oil (OPO). ^A–C^ Effect of formulation and cooling. Different letters in the same column indicate significant differences (*p* < 0.05).

**Table 4:** Performance of puff pastry (PP) prepared with margarines M1–M4 containing OPO in comparison with PP elaborated with controls CB and CFP.

PP	Weight Loss (%)	PP Lift (mm)	Specific Lift (mm/g)	Development (−)	Lift Irregularity (mm)	Shrinkage (−)
PP-CB	20.5 ± 1.3	44.1 ± 3.6	2.71 ± 0.33	7.61 ± 0.61	11.4	20.8 ± 7.4
PP-CFP	22.7 ± 3.2	40.1 ± 4.4	2.82 ± 0.45	6.92 ± 0.75	10.9	25.5 ± 11
PP-M1	23.2 ± 1.2 ^B^	30.1 ± 3.8 ^A^	1.73 ± 0.23 ^B^	5.84 ± 0.75 ^A^	11.5	19.0 ± 19 ^A^
PP-M2	23.1 ± 1.2 ^B^	23.6 ± 3.9 ^C^	1.32 ± 0.24 ^C^	4.46 ± 0.73 ^B^	15.6	28.0 ± 18 ^A^
PP-M3	25.7 ± 0.76 ^A^	27.4 ± 2.8 ^A,B^	1.98 ± 0.26 ^A^	4.94 ± 0.50 ^B^	18.0	21.6 ± 10 ^A^
PP-M4	25.6 ± 0.83 ^A^	24.8 ± 3.0 ^B,C^	1.74 ± 0.22 ^B^	4.40 ± 0.53 ^B^	11.6	17.8 ± 11 ^A^

Mean values (*n* = 13–18) ± standard deviation. PP-CB, PP made with a commercial butter; PP-CFB, PP made with a commercial fatty preparation; PP-M1, PP-M2, PP-M3, and PP-M4, PP prepared with four margarines containing olive pomace oil (OPO). ^A–C^ Effect of formulation and cooling. Different letters in the same column indicate significant differences (*p* < 0.05).

## Data Availability

Data is contained within the article or Supplementary Materials.

## Supplementary Materials

The following supporting information can be downloaded at: https://www.mdpi.com/article/10.3390/foods12112138/s1, Table S1. Formulation of four margarines (M1–M4) containing olive pomace oil (OPO) and cooling rate used for each one. Table S2. Fatty acid (FA) profile corresponding to olive pomace oil (OPO), palm stearin (PS), a puff pastry (PP) commercial butter (CB), a PP commercial fatty preparation (CFP) and two margarines containing OPO (M1 and M3). Figure S1. Stress sweeps carried out at 1 Hz and at 20 °C of a puff pastry (PP) commercial butter (CB), a PP commercial fatty preparation (CFP) and four margarines (M1–M4) containing olive pomace oil (OPO). Shear stress (*σ*) ranged between 20 and 2000 Pa. *G*′, elastic modulus; *G*″, viscous modulus. Figure S2. Frequency sweeps carried out at 1 Hz and at 20 °C to determine the mechanical spectra of a puff pastry (PP) commercial butter (CB), a PP commercial fatty preparation (CFP) and four margarines (M1–M4) containing olive pomace oil (OPO). *G*′, elastic modulus; *G*″, viscous modulus. Figure S3. Force–distance curves from a penetration test at 20 °C of a puff pastry (PP) commercial butter (CB), a PP commercial fatty preparation (CFP) and four margarines (M1–M4) containing olive pomace oil (OPO). Figure S4. Force–time curves from a texture analysis profile (TPA) test of a puff pastry (PP) made with a commercial butter (PP-CB), a PP made with a commercial fatty preparation (PP-CFP) and PP prepared with four margarines (PP-M1/PP-M4) containing olive pomace oil (OPO). Figure S5. Force–distance curves from a cutting test of a puff pastry (PP) made with a commercial butter (PP-CB), a PP made with a commercial fatty preparation (PP-CFP) and PP prepared with four margarines (PP-M1/PP-M4) containing olive pomace oil (OPO).
