# Supplementary material for: Functionality of Puff Pastry Olive Pomace Oil-Based Margarines and Their Baking Performance
Source: Foods. 2023 May 25;12(11):2138. doi: 10.3390/foods12112138 (PMC10252415; doi:10.3390/foods12112138)
Supplement: Supplementary file 1 [file foods-12-02138-s001.zip › foods-2413434-supplementary.pdf]

Table S1. Formulation of four margarines (M1-M4) containing olive pomace oil (OPO) and cooling rate used for each one.

Formulation	Cooling Rate (°C/min)	Aqueous Phase, AP (20%)						Fat Phase, FP (80%)									
		Water	Salt	Butter Flavor	Gelatine	Tartaric Acid	Citric Acid	OPO	Palm Stearin (PS)	Beeswax	Table Butter	Verolec Non GMO IP	Palsgaard 1311®	Cocoa Butter	Verol N-90	Verol P
M1	0.144	17.9	1.0	0.37	0.50	0.25	0.0025	40.8	23.5	3.0	7.5	1.5	1.17	-	0.83	1.75
M2	0.380	17.9	1.0	0.37	0.50	0.25	0.0025	40.8	23.5	3.0	7.5	1.5	1.17	-	0.83	1.75
M3	0.144	17.9	1.0	0.37	0.50	0.25	0.0025	30.8	23.5	3.0	7.5	1.5	1.17	10	0.83	1.75
M4	0.380	17.9	1.0	0.37	0.50	0.25	0.0025	30.8	23.5	3.0	7.5	1.5	1.17	10	0.83	1.75

Composition is expressed as percentage (%).

Table S2. Fatty acid (FA) profile corresponding to olive pomace oil (OPO), palm stearin (PS), a puff pastry (PP) commercial butter (CB), a PP commercial fatty preparation (CFP) and two margarines containing OPO (M1 and M3).

**Fatty Acids (mg FA/g Sample)**	**OPO**	**PS**	**CB**	**CFP**	**M1**	**M3**
Butyric C4:0	ND	ND	10.7±0.25	ND	0.703±0.027	0.716±0.029
Caproic C6:0	ND	ND	11.4±0.23	ND	0.800±0.013	0.812±0.018
Caprylic C8:0	ND	0.344±0.0045	9.0±0.12	ND	0.866±0.018	0.857±0.014
Capric C10:0	ND	0.317±0.0055	22.4±0.26	ND	2.06±0.011	2.04±0.012
Lauric C12:0	ND	3.60±0.012	30.2±0.32	0.359±0.011	3.40±0.00094	3.44±0.0079
Myristic C14:0	ND	10.5±0.028	94.5±1.1	3.22±0.066	10.9±0.031	10.9±0.030
Pentadecylic C15:0	ND	0.734±0.013	10.1±0.13	0.238±0.017	1.05±0.0080	1.10±0.0060
Palmitic C16:0	112±0.81	614±1.0	284±3.8	220±4.18	280±0.87	297±2.4
Margaric C17:0	0.767±0.024	1.24±0.0023	5.48±0.075	0.652±0.035	1.17±0.014	1.34±0.011
Stearic C18:0	26.7±0.22	67.7±0.123	84.3±1.2	34.0±0.55	56.5±0.12	97.2±0.54
Arachidic C20:0	4.49±0.072	3.75±0.026	1.08±0.066	8.43±0.13	3.81±0.029	4.61±0.020
Behenic C22:0	1.92±0.016	0.755±0.034	0.46±0.0053	4.96±0.024	1.50±0.0058	1.45±0.016
Lignoceric C24:0	0.857±0.030	0.739±0.027	ND	5.81±0.16	2.25±0.036	1.89±0.022
**∑SFA**	**147**	**704**	**563**	**278**	**354**	**403**
Palmitoleic C16:1n7	7.90±0.057	0.579±0.0049	13.5±0.16	4.40±0.094	5.24±0.041	4.62±0.0076
Vaccenic C18:1n7	19.9±0.17	3.06±0.099	3.87±0.072	11.5±0.26	11.7±0.082	9.66±0.024
Oleic C18:1n9	654±5.3	184±0.43	163±2.0	392±8.6	411±2.8	372±0.83
**∑MUFA**	**685**	**188**	**180**	**408**	**429**	**387**
Linoleic C18:2n6	108±0.84	35.8±0.14	13.1±0.56	76.7±1.7	73.5±0.51	63.7±0.14
Linolenic C18:3n3	7.18±0.061	0.482±0.0047	3.69±0.095	3.81±0.062	4.49±0.025	3.84±0.022
**∑PUFA**	**116**	**36.3**	**16.8**	**80.5**	**78.7**	**68.2**
**∑MUFA+∑PUFA**	**801**	**224**	**197**	**488**	**508**	**456**

Mean values (n = 3) ± standard deviation. ND, not detected. SFA, saturated fatty acids; MUFA, monounsaturated fatty acids; PUFA, polyunsaturated fatty acids.

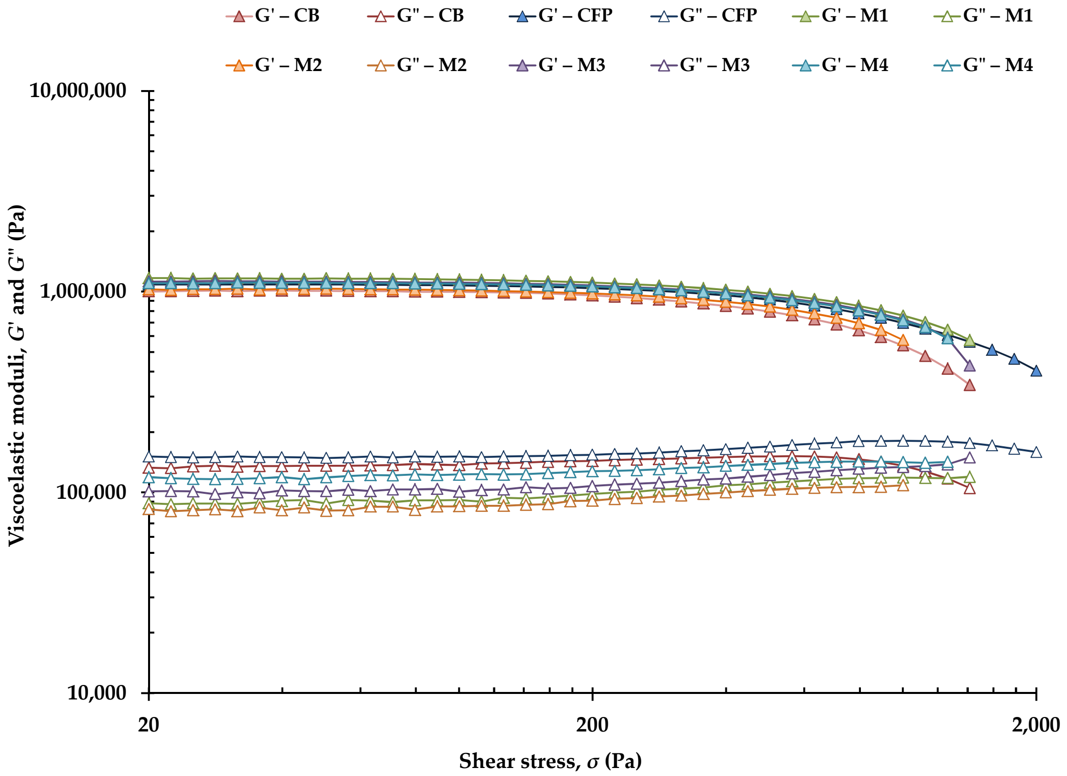

Figure S1. Stress sweeps carried out at 1 Hz and at 20 °C of a puff pastry (PP) commercial butter (CB), a PP commercial fatty preparation (CFP) and four margarines (M1-M4) containing olive pomace oil (OPO). Shear stress (σ) ranged between 20 and 2,000 Pa. G', elastic modulus; G'', viscous modulus.

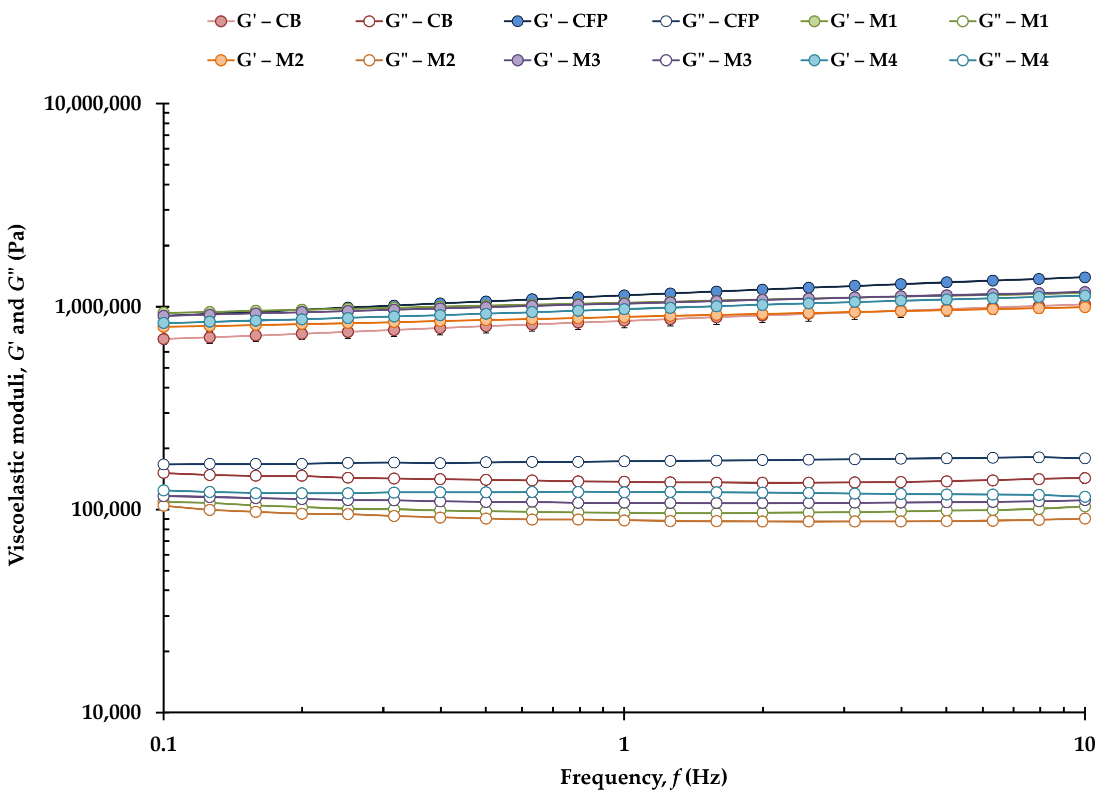

Figure S2. Frequency sweeps carried out at 1 Hz and at 20 °C to determine the mechanical spectra of a puff pastry (PP) commercial butter (CB), a PP commercial fatty preparation (CFP) and four margarines (M1-M4) containing olive pomace oil (OPO). *G*′, elastic modulus; *G*″, viscous modulus.

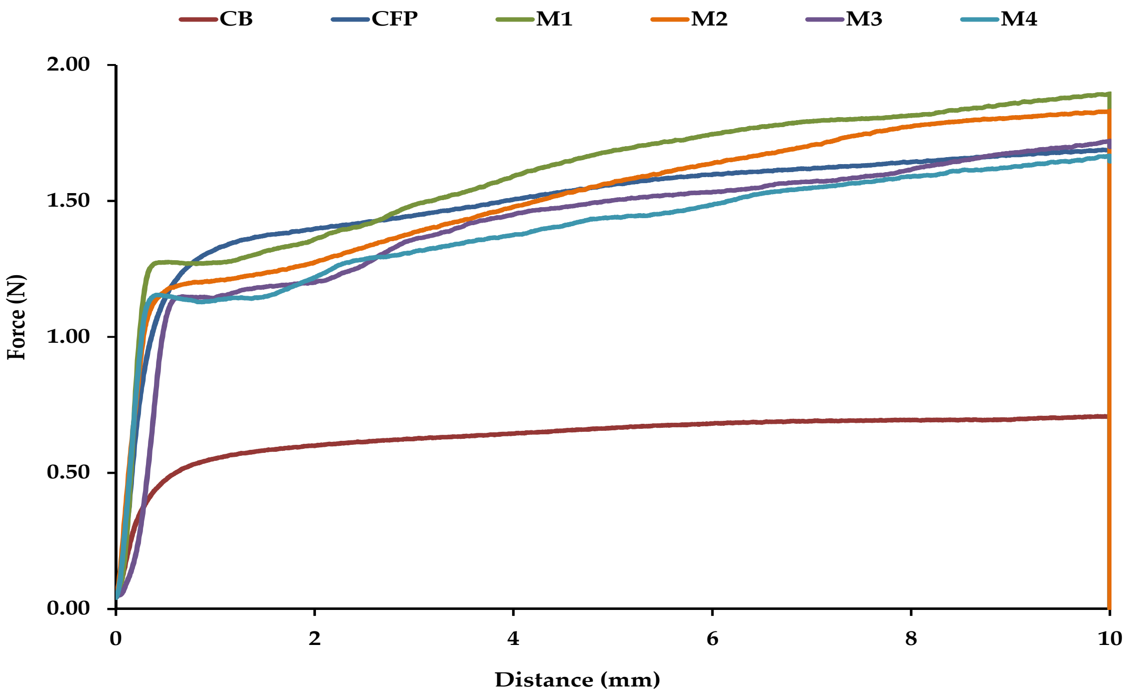

Figure S3. Force–distance curves from penetration test at 20 °C of a puff pastry (PP) commercial butter (CB), a PP commercial fatty preparation (CFP) and four margarines (M1-M4) containing olive pomace oil (OPO).

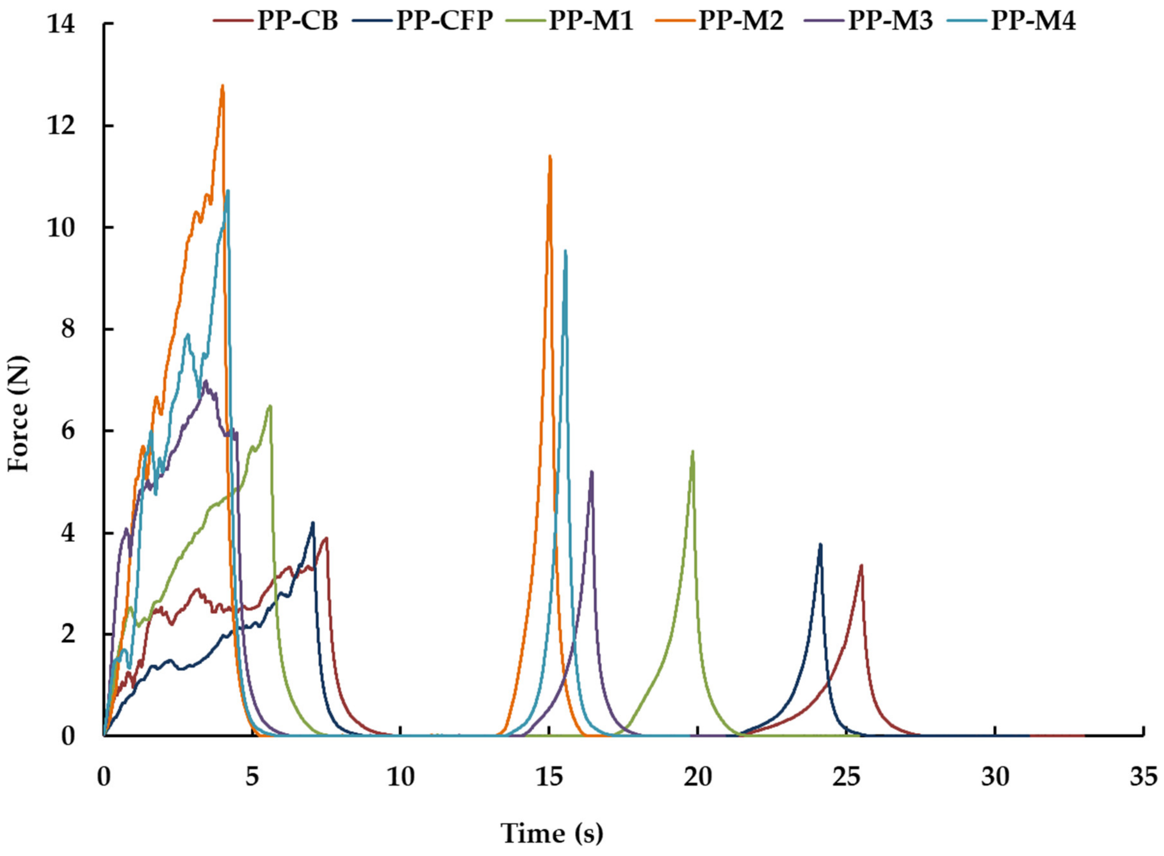

Figure S4. Force–time curves from a texture analysis profile (TPA) test of a puff pastry (PP) made with a commercial butter (PP-CB), a PP made with a commercial fatty preparation (PP-CFP) and PP prepared with four margarines (PP-M1/PP-M4) containing olive pomace oil (OPO).

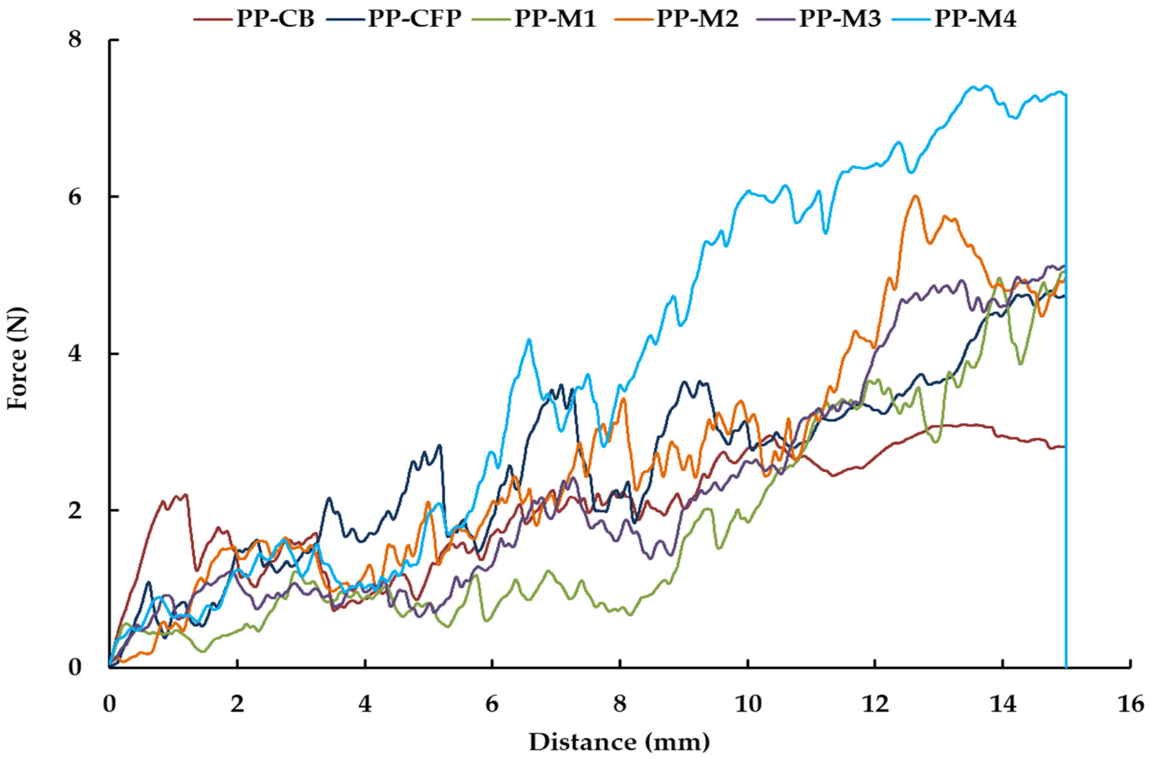

Figure S5. Force–distance curves from a cutting test of a puff pastry (PP) made with a commercial butter (PP-CB), a PP made with a commercial fatty preparation (PP-CFP) and PP prepared with four margarines (PP-M1/PP-M4) containing olive pomace oil (OPO).
